# Risky Sexual Behavior and Psychopathy: Testing the Relationship in a Non-Clinical Sample of Young Adults in Hong Kong

**DOI:** 10.3390/bs14020094

**Published:** 2024-01-27

**Authors:** Heng Choon (Oliver) Chan, Anthony Beech

**Affiliations:** 1 Department of Social Policy, Sociology, and Criminology, School of Social Policy, University of Birmingham, Birmingham B15 2TT, UK; 2 School of Psychology, University of Birmingham, Birmingham B15 2TT, UK

**Keywords:** risky sexual behavior, psychopathy, psychopathic traits, psychosocial risk factors, young adults, Hong Kong

## Abstract

This study aims to investigate the relationship between risky sexual behavior (RSB) and psychopathy in a non-clinical sample of 714 Hong Kong adults, shedding light on sex differences. Our findings reveal that males exhibit significantly higher mean levels of RSB (general, penetrative, and nonpenetrative), as well as egocentric (Factor 1) and callous (Factor 3) traits of psychopathy, along with increased sexual desire compared to females. Regression analyses indicate that elevated levels of antisociality (Factor 2) and callousness (Factor 3) traits of psychopathy, along with sexual desire, emerge as significant risk factors for engaging in general, penetrative, and nonpenetrative RSB. Intriguingly, higher age and being in a long-term relationship are associated with RSB. The implications of this study suggest potential avenues for reducing, if not entirely preventing, the inclination to engage in RSB in the presence of psychopathic traits.

## 1. Introduction

Risky sexual behavior (RSB) is a global public health concern, and its consequences impact many people each year. Examples of RSB include unprotected sexual intercourse and inconsistent or inaccurate use of contraception (e.g., condom use). Additionally, the use of any illicit drug just before or during planned sex to facilitate, initiate, prolong, sustain, and intensify the encounter, generally referred to as ‘chemsex’, is commonly observed in the homosexual population, especially men who have sex with men [1]. The Global Burden of Disease Study (1990–2013) reported that unsafe sexual practices by young people (aged 10–24 years) from 188 nations is a contributing factor for an increased level of disability-adjusted life-years (i.e., the total years of potential life lost because of premature death and the years of productive life lost from disability) [2]. The World Health Organization [3] reported that more than a million people are infected with a sexually transmitted infection (STI), such as Chlamydia trachomatis, gonorrhea, syphilis, and HIV infections, each day. In addition to these STIs, active engagement in RSB can result in poor long-term reproductive health outcomes, such as unintended pregnancy, infertility, and pelvic inflammatory disease [4,5].

Numerous studies in different countries (e.g., Addis Ababa, Thailand, and the U.S.) have reported that a heightened risk for negative sexual health outcomes was found among adolescents and young adults partly due to their frequent practice of unprotected sex with multiple partners [6,7,8,9]. In Hong Kong, a household survey of 881 young and middle-aged adults (aged 18–49 years) found a higher prevalence rate of Chlamydia trachomatis (5.8%) among sexually active young females (18–26 years), although a low overall rate (1.4%) was observed for the full sample [10]. However, mixed findings have been found on sex differences in RSB. Although many studies reported that males tended to engage in more RSB than females [e.g., 11,12,13], some studies have either reported that females practice more RSB than males [14] or have found no significant sex difference [15].

Similar to RSB, psychopathy is a widely studied psychopathology in criminology and psychology. Psychopathy is a clinical construct characterized by a pattern of behavioral, affective, and interpersonal traits, such as callousness, impulsivity, grandiosity, lack of empathy, and a range of antisocial actions [16,17,18]. Most conceptualizations of psychopathy have been either personality- or behavior-based [19]. Hare [20] argued that psychopathic individuals are unlikely to change fundamentally with age. However, they may be involved in different types of antisocial behavior across their lifespan [20]. Psychopathy is rare among nonoffenders, accounting for about 1% of the general population [21,22,23], whereas the prevalence in offender populations is much higher (e.g., 15–25% of male offenders in the Canadian federal correctional system) [20,24].

Relevant to this study, the prevalence of psychopathy among sex offenders varies from 8% to 35%, depending on the forensic population sampling; for example, reported rates range between 8% [25,26], 9.9% [27], 15% [28], 29% [29], and 35% [30]. In part, prevalence rates vary due to different levels of psychopathy being used to diagnose an individual being psychopathic. For example, using the traditional Hare Psychopathy Checklist-Revised (PCL-R) cutoff score of 30 or greater, Olver and Wong [25] found that 8% of their sample met the criteria for psychopathy. However, when they adopted a cutoff score of 25 or greater, the prevalence of psychopathy was 19%.

Psychopathy has been proven to be a robust predictor of antisocial, aggressive, and offending behavior, especially in relation to violence [e.g., 31,32,33]. Notably, psychopathy is deemed to be correlated with RSB, such as engaging in promiscuous sexual relations at a young age for both males and females [34] and sexually impulsive and irresponsible behavior in female adolescents [35]. From the neurocognitive perspective, Hughes et al. [36] stated that an underactive inhibition system may result in difficulty in regulating behavior, including failure to inhibit unwanted sexual activity in potentially threatening contexts. Therefore, individuals who are impulsive and thrill-seeking tend to engage in risky sexual endeavors, such as engaging in unprotected sexual activities, having multiple sexual partners, and being involved in high-risk encounters (e.g., drugs and/or alcohol intoxication, having casual partners) [5]. Similarly, Gottfredson and Hirschi [37] postulated in their self-control theory that individuals who are low in self-control are more likely to be impulsive, risk-seeking, self-centered, short-tempered, and prefer to choose simple tasks (over complex tasks) and physical activities (over mental activities). They are more likely than those who are less impulsive to become involved in deviant (e.g., risky) and criminal activities, searching for immediate satisfaction.

Most studies have found that males generally possess more psychopathic traits than females using both clinical measures of forensic populations [e.g., 38,39,40,41] and self-reported measures of community populations [e.g., 42,43]. However, other studies have failed to find any significant sex differences, especially in psychopathy correlates or factor structure [e.g., 44,45]. Thus, it remains open to debate whether the observed differences in the occurrence of male and female psychopaths reflect actual physical differences in the frequency of psychopathy or whether the differences are due to the differential in diagnostic tools and the terminology used, which may be indicated when these criteria for assessing psychopathy are applied to females [46,47,48].

Relevant to this study, understanding the relationship between psychopathy and RSB remains important from both public health and criminal justice perspectives; for instance, practicing RSB may lead to committing such behavior as nonconsensual sexual activities. Given the potential high risk of negative public health and criminal justice outcomes, it is worthwhile studying this phenomenon to provide a better understanding of such behavior in Hong Kong. Timely identification and strategized intervention are essential to prevent RSB and its possible escalation to more serious and chronic deviant sexual behavior (e.g., sexual assault and rape). More importantly, as most studies have been conducted with Western samples, this study adds geographical diversity by investigating an under-researched population, viz., Hong Kong adults.

## 2. Risky Sexual Behavior (RSB) and Psychopathy

Personality features, including impulsivity, low self-control, sensation seeking, and low agreeableness, have consistently been found to be predictive of RSB [5,49]. Hoyle et al. [5] found that impulsivity and low conscientiousness are personality characteristics often associated with practicing unprotected sex (i.e., RSB). These maladaptive personality traits are also factors that are integral to psychopathy [50]. Individuals with a high psychopathic level are characterized by a host of maladaptive personality traits, including interpersonal and affective dysfunction (e.g., impulsivity, manipulativeness, and lack of remorse) [51,52,53]. Traditionally, psychopathy has been evaluated through clinical interviews, with the Hare PCL-R being the most commonly adopted instrument. The PCL-R is an expert-administered rating scale comprising a semi-structured interview (20 items scored on a 3-point scale [0, 1, 2]) and a review of collateral information, including official criminal justice files and medical records [52,54]. Although PCL-R is widely used in measuring psychopathy, the self-reported approach is not uncommon in assessing psychopathic traits among non-forensic populations.

A study related to this one that investigated the association between RSB and psychopathic traits in a sample of college students found that self-reported psychopathy scores, measured using the Self-Report Psychopathy Scale-III [55], were positively associated with the number of sexual partners [56]. In another study of college students, Fulton et al. [57] found that higher scores on the Fearless Dominance (FD) and Impulsive Antisociality (IA) subscales of the Psychopathic Personality Inventory [44] were positively correlated with RSB, as measured by number of sexual partners, condom usage, and having sexual activities while intoxicated (based on the Sexual Risk Survey) [58]. A further study that recruited college students, Kastner and Sellbom [59] found that FD and IA scores were strong predictors of hypersexuality compared with other factors, including sensation seeking, impulsivity, and antisociality. Similarly, evidence of a positive relationship between RSB and psychopathy has been found in studies that recruited other sampling populations, including community adolescents [35,60,61], incarcerated juvenile offenders [62], incarcerated male adult offenders [63], and incarcerated female offenders [64].

## 3. The Study

Hong Kong, a special administrative region of the People’s Republic of China (PRC), had a population of 7.41 million people in 2021 [65]. About 95% of its occupants are of Chinese descent, and the official languages are English and Chinese Cantonese. As a former British colony for more than 150 years, Hong Kong residents largely balance their modern Western lifestyles with traditional Chinese cultural values and practices.

Although positive correlations between RSB and psychopathy have been found in most studies conducted in the West, this relationship remains unclear in a Chinese cultural context, particularly in the Hong Kong population. Compared with Western cultures, Asian and Middle Eastern cultures tend to have more restrictive perspectives on sexual issues, with a discussion of sexual activity having long been considered taboo in these cultures [66,67]. Nonetheless, attitudes and values regarding sexual interests, activity, and sexuality are changing as societies evolve, change, and take on new customs [68]. Against this backdrop, the importance of this study is twofold. First, it is probably the first to investigate the relationship between RSB and psychopathy in a community sample of young adults in Hong Kong. Second, the findings of this study we suggest can inform practice (e.g., preventive and intervention measures) through the identification of significant risk factors (e.g., psychopathy) that predispose an individual to engage in different types of RSB. Timely and effective interventions can reduce the likelihood of young adults engaging in RSB, who may run the risk of progressing to more criminally oriented RSB (e.g., nonconsensual sexual activities). There are two proposed hypotheses.

**Hypothesis** **1:** *There are sex differences in relation to RSB (i.e., general, penetrative, and nonpenetrative behavior), self-reported psychopathy (i.e., general psychopathy, and egocentric, antisocial, and callous traits, which are Factors 1, 2, and 3, respectively), and psychosocial risk factors (i.e., impulsivity and sexual desire), such that males are anticipated to have higher mean levels of RSB, psychopathy, impulsivity, and sexual desire than females.*

**Hypothesis** **2:** *Self-reported psychopathy is correlated with all types of RSB, even when accounting for demographic characteristics (i.e., age, sex, religiosity, and intimate relationship status) and psychosocial risk factors (i.e., impulsivity and sexual desire), such that a high level of psychopathy is correlated with the participants’ likelihood of engaging in all RSB types.*

## 4. Methods

### 4.1. Procedure

Seven hundred and fourteen participants, aged 18 years and above, were recruited from all eight public (i.e., government-funded) universities and three private universities in Hong Kong. The participants were invited to participate in the questionnaire survey after being randomly approached within the university environs (e.g., libraries, reading corners, and student cafeterias) or through a convenience sampling method (recruitment from classrooms with prior consent from the lecturers and via word-of-mouth among participants). The first method accounted for about 55% of participants recruited, and the latter for about 45%.

The participants were given the option of completing a paper-and-pen survey or an online survey developed through Qualtrics (a tool used for creating and distributing questionnaire surveys). Around 80% chose the online survey, with the rest completing the paper-and-pen version. Prior to the administration of the survey, the participants’ informed consent was obtained, with assurances given that their responses would be kept confidential and anonymous. Their participation was voluntary, and no monetary incentives were involved. On average, the participants took 25 min to complete the survey in private without any interruption. The response rate was about 90%.

### 4.2. Participants

Three-quarters of the participants were Hong Kong residents (77.2%), with the remaining being mainland Chinese (15.3%) and other nationalities (7.6%). Females constituted 69.5% of the sample, and 30.5% were males. The mean age of all participants was 20.62 years (*SD* = 2.89, range = 18–40 years; see Table 1), with the mean ages of female and male participants being 20.41 years (*SD* = 2.54) and 21.09 years (*SD* = 3.53), respectively. Two-thirds of the participants were single (66.5%), slightly over half reported that they were post-secondary school educated (55.2%), and nearly three-quarters had no religious affiliation (70.2%).

### 4.3. Ethical Considerations

This study was approved by the Human Subjects Ethics Sub-Committee of the first author’s university. At any time, the participants could end their participation, contact the primary investigator, and/or receive professional counseling. The authors take responsibility for the integrity of the data and the accuracy of the data analyses and have made every effort to avoid inflating statistically significant results.

### 4.4. Measures

In addition to the sociodemographic questionnaire (e.g., age, sex, country of origin, education, intimate relationship status, and religious belief), four measures were used to explore (a) sex differences in the general and subtypes of RSB and psychopathy, impulsivity, and sexual desire; (b) the relationship between the general and subtypes of RSB and psychopathy; and (c) the effects of psychopathy and psychosocial risk factors on general, penetrative, and nonpenetrative RSB. These were prepared in both English and Chinese to accommodate the participants’ varying language abilities. The back-translation approach (i.e., English-to-Chinese initially and subsequently Chinese-to-English) was adopted to ensure the validity and consistency of the translated measures.

#### 4.4.1. Sexual Risk Survey

This 23-item survey [58] was adopted to measure the participants’ level of involvement in RSB over the past six months. The scoring of the Sexual Risk Survey items was dichotomized (0 = no, 1 = yes) and consisted of two subscales on penetrative (16 items) and nonpenetrative RSB (7 items), respectively), with a total score of 0 to 23. A higher (lower) score indicated a greater (lesser) involvement in RSB. Sample items asked whether the participants “Had vaginal intercourse without protection against pregnancy” (penetrative RSB) and “Had ‘hooked up’ but not had sex with someone you didn’t know or didn’t know well” (nonpenetrative RSB). Cronbach’s α of this measure was 0.93 (males = 0.94, females = 0.91); the alpha coefficients of the penetrative subscale was 0.91 (males = 0.93, females = 0.89), for nonpenetrative RSB 0.80 (males = 0.84, females = 0.76).

#### 4.4.2. Levenson’s Self-Report Psychopathy Scale

This psychopathy measure comprised 26 items and was scored on a 4-point Likert scale (1 = strongly agree, 4 = strongly disagree). The total score ranged from 26 to 104, with a higher (lower) score denoting a more (less) psychopathic personality. The three-factor structure proposed by Brinkley et al. [69] was used: (1) Factor 1—egocentric (10 items), (2) Factor 2—antisocial (5 items), and (3) Factor 3—callous (4 items). Seven items that were not loaded clearly on one factor were excluded. It has been found [69] that this three-factor structure fits the data better than the original two-factor model of Levenson et al. [70], and it has been successfully replicated in other studies, including in Bulgaria [71], in the U.S. [72], in the U.K. [73], in Italy [74], and mainland China [75,76], and with both prison- and community-based samples. Sample items include “Success is based on survival of the fittest; I am not concerned about the losers” (Factor 1), “I find myself in the same kinds of trouble, time after time” (Factor 2), and “I make a point of trying not to hurt others in pursuit of my goal” (reverse scored, Factor 3). Cronbach’s α was 0.72 (males = 0.76, females = 0.72), while the alpha coefficients of the subscales were 0.75 for Factor 1 (males = 0.80, females = 0.71), 0.70 for Factor 2 (males = 0.70, females = 0.70), and 0.65 for Factor 3 (males = 0.73, females = 0.62).

#### 4.4.3. Impulsivity Subscale of the Low Self-Control Scale

The Low Self-Control Scale has been consistently found to yield good construct validity in assessing the low self-control indicators and has high reliability across gender and geographical populations (e.g., North American, European, and Asian samples) [e.g., 77,78,79,80]. Impulsivity was measured on a 4-point Likert scale (1 = strongly agree, 4 = strongly disagree), with a total score ranging from 4 to 16. A higher score indicated greater impulsivity (or weaker impulse control), and vice versa. Sample items include “I often act on the spur of the moment without stopping to think” and “I always do whatever brings me pleasure here and now, even at the cost of some distant goal”. The Cronbach’s α of this measure was 0.60 (males = 0.68, females = 0.58).

#### 4.4.4. Sexual Desire Inventory

Level of sexual desire was measured with a 14-item Sexual Desire Inventory using a 5-point Likert scale (0 = no desire, not at all important, or much less desire, 4 = strong desire, extremely important, or much more desire), with a total score ranging from 0 to 56 [81]. A higher (lower) score denoted greater (lesser) sexual desire. Sample items asked the participants, “When you have sexual thoughts, how strong is your desire to engage in sexual behavior with a partner” and “When you are in romantic situation, how strong is your sexual desire”. The Cronbach’s α of this measure was 0.93 (males = 0.89, females = 0.92).

## 5. Data Analytic Strategy

Independent sample *t*-tests were first performed to explore the difference between sexes in terms of the different types of RSB (i.e., general, penetrative, and nonpenetrative behavior), self-reported psychopathy (general and Factors 1 to 3), impulsivity, and sexual desire. Pearson correlations were computed to explore the interrelatedness of the general and subtypes of RSB and psychopathy. The Bonferroni correlation was performed to reduce the instance of a false positive in analysis with multiple comparisons. This statistical adjustment was to prevent data from incorrectly appearing to be statistically significant. Finally, ordinary least squares (OLS) regressions were conducted to explore the effects of self-reported psychopathy (with Model I assessing general psychopathy and Model II assessing the three-factor structure) and psychosocial risk factors (i.e., impulsivity and sexual desire) on different types of RSB (i.e., general, penetrative, and nonpenetrative behavior), while accounting for the participants’ demographic characteristics (i.e., age, sex, religiosity, and intimate relationship status). The participants’ religiosity was measured by how religious they think they are on a 6-point Likert scale (1 = not at all, 6 = very strongly). Pearson correlations of the variables were computed. No correlation at or above 0.70 was noted, indicating no collinearity. The check on normality and homoscedasticity were performed, and no violation of the assumption was found. The significance level was set at *p* < 0.05.

## 6. Results

### 6.1. Sex Differences in the Means of RSB, Psychopathy, and Psychosocial Risk Factors

Table 2 presents the mean differences between the male and female participants’ levels of self-reported RSB, psychopathy, impulsivity, and sexual desire. Several significant sex differences were found. Compared with the female participants, the male participants reported significantly higher levels of general (*t* = 3.35, *p* < 0.001), penetrative (*t* = 3.19, *p* = 0.002), and nonpenetrative (*t* = 3.07, *p* = 0.002) RSB. Males also reported significantly higher levels of Factor 1—egocentric (*t* = 4.19, *p* < 0.001), and Factor 3—callous (*t* = 1.87, *p* = 0.031) subtypes of psychopathy, and high levels of sexual desire (*t* = 10.66, *p* < 0.001) compared with females. No significant sex differences were observed in the participants’ general psychopathy, Factor 2—antisocial subtype of psychopathy, or impulsivity.

### 6.2. Pearson Correlations of RSB and Psychopathy

Table 3 shows the Pearson correlations examining the relationships between the general and subtypes of RSB and psychopathy. Significant positive correlations were observed after Bonferroni adjustments. The correlation coefficients for the total sample ranged from 0.09 to 0.97 (10 significant correlations); for the male participants, they ranged from 0.16 to 0.98 (8 significant correlations), and for the female participants, from 0.08 to 0.97 (10 significant correlations).

### 6.3. Effects of Psychopathy and Psychosocial Risk Factors on General, Penetrative, and Nonpenetrative RSB

OLS regressions were conducted to examine the effects of self-reported psychopathy and psychosocial risk factors on general, penetrative, and nonpenetrative RSB while accounting for the participants’ demographic characteristics (i.e., age, sex, religiosity, and intimate relationship status). The findings, shown in Table 4, indicate that the results for both Model I (general psychopathy) and Model II (subtypes of psychopathy) were significant. Although general psychopathy had no significant effect on general RSB, it was found that Factor 2 (antisocial personality) (*B* = 0.17, *SE* = 0.08, *p* = 0.03) and Factor 3 (callous personality) (*B* = 0.22, *SE* = 0.07, *p* < 0.001) psychopathy were positively associated with the participants’ propensity to engage in general RSB. Furthermore, being older (an increase in age; Model I: *B* = 0.16, *SE* = 0.06, *p* = 0.002; Model II: *B* = 0.17, *SE* = 0.06, *p* = 0.004), non-single (Model I: *B* = −1.89, *SE* = 0.37, *p* < 0.001; Model II: *B* = −1.85, *SE* = 0.36, *p* < 0.001), and having an increased level of sexual desire (Model I: *B* = 0.11, *SE* = 0.02, *p* < 0.001; Model II: *B* = 0.10, *SE* = 0.02, *p* < 0.001) were significant predictors of engaging in general RSB.

Although, again, general psychopathy was not found to be significantly correlated with penetrative RSB, Factor 2 (antisocial personality) (*B* = 0.14, *SE* = 0.06, *p* = 0.012) and Factor 3 (callous personality) (*B* = 0.19, *SE* = 0.05, *p* < 0.001) psychopathy traits were positively associated with the participants’ propensity to engage in penetrative RSB. Similarly, being older (Model I: *B* = 0.15, *SE* = 0.04, *p* < 0.001; Model II: *B* = 0.14, *SE* = 0.04, *p* = 0.001), non-single (Model I: *B* = −1.52, *SE* = 0.27, *p* < 0.001; Model II: *B* = −1.50, *SE* = 0.27, *p* < 0.001), and possessing an increased level of sexual desire (Model I: *B* = 0.08, *SE* = 0.01, *p* < 0.001; Model II: *B* = 0.07, *SE* = 0.01, *p* < 0.001) were significantly correlated with the participants’ tendency to participate in penetrative RSB. Notably, no significant relationship was found between the general and subtypes of psychopathy and nonpenetrative RSB. Nonetheless, being non-single (Model I: *B* = −0.36, *SE* = 0.13, *p* = 0.005; Model II: *B* = −0.36, *SE* = 0.13, *p* = 0.006) and having an increased level of sexual desire (Model I: *B* = 0.03, *SE* = 0.01, *p* < 0.001; Model II: *B* = 0.03, *SE* = 0.01, *p* < 0.001) were significantly associated with the likelihood of engaging in nonpenetrative RSB. Interestingly, the participants’ impulsivity was positively correlated with nonpenetrative RSB in Model II (*B* = 0.05, *SE* = 0.03, *p* = 0.049).

## 7. Discussion

It has consistently been found that maladaptive personality traits, as manifested by psychopathic traits (e.g., callousness, impulsivity, lack of remorse, manipulativeness, and superficial charm), are positively associated with deviant and criminal sexual offending, including RSB [5,51,52,53]. This study is pertinent not only for its contribution to the growing literature on the relationship between RSB and psychopathy but also, more importantly, for its emphasis on a rarely researched population. Examining a community sample of 714 young adults aged 18 to 40 years, this study has two primary purposes: (1) to examine the mean differences of male and female participants in different RSB types (i.e., general, penetrative, and nonpenetrative) and self-reported psychopathy (i.e., general, Factor 1—egocentric, Factor 2—antisocial, and Factor 3—callous); and (2) to explore the relationship between different types of RSB and self-reported psychopathy when accounting for demographics (i.e., age, sex, religiosity, and intimate relationship status) and psychosocial characteristics (i.e., impulsivity and sexual desire). As a whole, males reported significantly more general, penetrative, and nonpenetrative RSB than females. Even though there was no significant sex difference in general psychopathy, the male participants reported having significantly higher mean scores than the female participants in Factor 1—egocentric and Factor 3—callous traits of psychopathy. These significant sex differences were consistent with most studies with non-clinical samples [e.g., 11,12,13,42,43; for a review of this literature, see 46].

There were some findings that deserve further discussion. Although general psychopathy failed to significantly correlate with all RSB types (i.e., general, penetrative, and nonpenetrative behavior), high levels of the antisocial (Factor 2) and callous (Factor 3) traits of psychopathy were reported to be significantly associated with general and penetrative RSB. This positive relationship aligns with the literature based on samples from other countries, including Canada, Croatia, the U.K., and the U.S. [34,35,36,56,57,82]. It is noteworthy that hypersexuality (e.g., RSB) has long been a defining feature of psychopathy and that it is often associated with antisocial and violent behavior [33,83], including sexual violence and sexual homicide [84,85,86]. Research has demonstrated that a high level of RSB is significantly correlated with sexual offending behavior [87] and that sexual deviancy is the strongest risk factor for sexual offending recidivism [88].

In addition, our study indicated that strong sexual desire was a significant predictor of all types of RSB. Sexual desire is the sum of the mental forces that lead an individual toward or away from engaging in actual sexual behavior [89,90]. High sexual desire can subsequently lead to high sexual arousal (i.e., physiological sexual reaction, e.g., getting and sustaining an erection), which can result in hypersexuality or compulsive sexual addiction [91]. This finding is aligned with the literature demonstrating that high sexual desire (e.g., sexual addiction, compulsive sexual behavior) is positively associated with involvement in RSB (e.g., condomless sex, soliciting prostitutes, frequenting strip clubs, and having casual sex partners) [92,93]. Individuals with high sexual desire (and/or sexual arousal) are likely to be high in sexual sensation seeking and thus willing to become involved in risky sexual situations/conditions (e.g., risking the possibility of being infected with STIs).

Demographically, our findings demonstrated that older (younger) participants were more (less) likely to engage in general and penetrative RSB and that participants who were non-single were significantly associated with all types of RSB. Some studies have reported that older individuals [e.g., 9,94] and non-single individuals, such as married/cohabiting couples, in a long-term intimate relationship [e.g., 95,96] are more likely to have an increased propensity to engage in RSB. Pinyopornpanish et al. [9] reasoned that it is possible that older individuals are more likely to be married and/or have a regular partner than younger individuals, and hence, they are less likely to engage in protected sex (e.g., regular condom use). Nonetheless, more empirical studies are required to unveil the complexity of this relationship.

Several limitations must be acknowledged. First, this was a cross-sectional study, and thus, the findings can only be interpreted in correlational terms. In future studies, a longitudinal approach can be used to improve understanding of the causal relationships between RSB and psychopathy, especially in the presence of potential psychosocial risk factors other than those examined in this study (e.g., adverse childhood maltreatment and drug and alcohol use). Next, as the present study only analyzed self-reported data, biases such as retrospective recall and social desirability are possible to have influenced the participants’ honesty in reporting their attitudes, perceptions, and experiences. Hence, a response bias measure could be used to reduce the probability of reporting biases in future research. Lastly, the sample recruited was from universities, and two-thirds of the participants were females, which may have diminished the generalizability of the study to a broad population. Therefore, future studies can consider recruiting a larger sample size with a diverse range of participants. However, the sample in this study can be considered representative of the wider population of Hong Kong university students, as the participants were recruited from all of the universities in Hong Kong.

### Implications of the Findings

The adverse outcomes of psychopathic traits and probable escalation of RSB to more serious and chronic behavior should not be overlooked. In addition to the potentially severe outcomes of RSB (e.g., sexual offending) that have been found consistently in the literature, it has been argued that psychopathy is the single most reliable predictor of criminal behavior and reoffending for both genders [97]. The findings of this study have various implications. By identifying specific risk factors for engaging in RSB, including scoring high on self-reported psychopathy (e.g., antisocial and callous personality traits), individuals with elevated psychopathic traits may be identified as benefiting from sex education and prevention programs. For instance, public awareness of the severity outcomes of RSB, such as a high risk of contracting STIs (e.g., HIV), should be enhanced. When targeting late adolescents and young adults, efforts should be prioritized much earlier than their life stage of sexual exploration. For example, Hong Kong has consistently faced criticism for the lack of comprehensive and effective school-based sexual education [98]. Thus, in addition to a community awareness campaign, it is especially pertinent that school/university prevention programs incorporate educational materials on the risks associated with the premature onset of sexual activities and sexually deviant activities (e.g., unprotected sex, multiple sexual partners). The content of such prevention programs should aim for specific behavioral changes, such as cultivating attitudes toward safe and socially acceptable sexual practices (e.g., consensual and noncoercive sexual activities), negotiating condom use for safer sex, and reducing the number of sexual partners. Latka et al. [99] reported that providing individuals with counseling on how to improve their protection from STIs, including condom use education, led to significant increases in their use of condoms in future sexual encounters.

Furthermore, issues relevant to the role of psychopathy in the development of RSB (e.g., victim empathy) should be addressed. For example, it is likely that the development of values for specific norms (e.g., care-based and fairness norms) is compromised for individuals with callous–unemotional traits because they tend not to find the distress of other individuals aversive [100]. Public mental health seminars that promote prosocial behavior such as victim empathy, unselfishness, and anger management can be helpful in disseminating practical information to target the antisocial and callous traits of psychopathy. In targeting young people who are at risk or practicing RSB, social norm interventions can be employed to address misperceptions about healthy sexual experiences. Irrespective of the forms of prevention and intervention programs, the development and delivery of such programs should be culturally sensitive, as Western practices may not be entirely applicable in the Asian context.

## Figures and Tables

**Table 1:** Sample demographic characteristics (*N* = 714).

Demographic Characteristics	*N*	Percentage
Sex		
Male	218	30.5%
Female	496	69.5%
Country of origin		
Hong Kong	551	77.2%
Mainland China	109	15.3%
Others	54	7.6%
(e.g., South Korea, Indonesia, Germany, The Netherlands, Taiwan, U.S.)		
Intimate relationship status		
Single	475	66.5%
Non-single	239	33.5%
Highest education attainment		
Secondary school education	320	44.8%
Post-secondary school education	394	55.2%
(e.g., associate degree/high diploma and undergraduate and postgraduate degrees)		
Religious belief		
Without a religious belief	501	70.2%
With a religious belief	213	29.8%
(e.g., Christianity, Catholic, Buddhism, Muslim)		

**Table 2:** Sex differences in the prevalence of self-reported risky sexual behavior, psychopathy, impulsivity, and sexual desire.

	All Sample		Male		Female		
Variables	(*N* = 714)		(n = 218)		(n = 496)		*t* Value
	*M*	*SD*	*M*	*SD*	*M*	*SD*	
Risky sexual behavior							
General behavior	3.11	4.75	4.11	5.78	2.66	4.14	3.35 ***
Penetrative behavior	2.14	3.50	2.84	4.23	1.83	3.08	3.19 **
Nonpenetrative behavior	0.96	1.59	1.27	1.90	0.83	1.41	3.07 **
Self-report psychopathy							
General psychopathy	68.06	8.67	68.85	9.02	67.72	8.50	1.61
Factor 1—Egocentric	24.01	4.62	25.16	5.02	23.51	4.35	4.19 ***
Factor 2—Antisocial	11.94	2.49	12.02	2.46	11.90	2.51	0.58
Factor 3—Callous	7.25	2.47	7.51	2.10	7.13	2.61	1.87 *
Psychosocial risk factors							
Impulsivity	10.54	2.48	10.46	2.10	10.58	2.64	−0.60
Sexual desire	23.91	12.01	30.38	10.32	21.08	11.60	10.66 ***

* *p* < 0.05, ** *p* < 0.01, *** *p* < 0.001.

**Table 3:** Pearson correlations of self-reported risky sexual behavior and psychopathy.

RSB and Psychopathy	GRSB	PRSB	NRSB	GP	F1	F2	F3
All sample (*N* = 714)							
General RSB (GRSB)							
Penetrative RSB (PRSB)	0.97 **						
Nonpenetrative RSB (NRSB)	0.85 **	0.70 **					
General psychopathy (GP)	0.05	0.03	0.10				
Factor 1—Egocentric (F1)	0.02	0.02	0.09	0.83 **			
Factor 2—Antisocial (F2)	0.09	0.12 *	0.01	0.67 **	0.48 **		
Factor 3—Callous (F3)	0.18 **	0.20 **	0.10	0.34 **	0.01	0.09	
Male (n = 218)							
General RSB (GRSB)							
Penetrative RSB (PRSB)	0.98 **						
Nonpenetrative RSB (NRSB)	0.88 **	0.75 **					
General psychopathy (GP)	0.03	0.01	0.09				
Factor 1—Egocentric (F1)	0.01	0.04	0.11	0.87 **			
Factor 2—Antisocial (F2)	0.05	0.09	0.05	0.65 **	0.47 **		
Factor 3—Callous (F3)	0.16	0.20 *	0.05	0.26 **	0.02	0.17	
Female (n = 496)							
General RSB (GRSB)							
Penetrative RSB (PRSB)	0.97 **						
Nonpenetrative RSB (NRSB)	0.82 **	0.65 **					
General psychopathy (GP)	0.04	0.02	0.08				
Factor 1—Egocentric (F1)	0.01	0.03	0.05	0.81 **			
Factor 2—Antisocial (F2)	0.12	0.15 *	0.02	0.68 **	0.49 **		
Factor 3—Callous (F3)	0.19 **	0.19 **	0.12	0.36 **	0.03	0.07	

Note: Bonferroni corrected. * *p* < 0.005, ** *p* < 0.001.

**Table 4:** OLS regression models of self-reported psychopathy and psychosocial risk factors on risky sexual behavior.

	General Behavior		Penetrative Behavior		Nonpenetrative Behavior	
Variables	Model I	Model II	Model I	Model II	Model I	Model II
	*B* (*SE*)	*B* (*SE*)	*B* (*SE*)	*B* (*SE*)	*B* (*SE*)	*B* (*SE*)
Demographic characteristics						
Age	0.18 (0.06) **	0.17 (0.06) **	0.15 (0.04) ***	0.14 (0.04) **	0.03 (0.02)	0.03 (0.02)
Sex	0.10 (0.38)	0.08 (0.38)	0.04 (0.28)	0.04 (0.28)	0.06 (0.13)	0.04 (0.13)
(0 = female, 1 = male)						
Religiosity	−0.02 (0.11)	−0.01 (0.11)	0.02 (0.08)	0.03 (0.08)	−0.03 (0.04)	−0.03 (0.04)
Intimate relationship status	−1.89 (0.37) ***	−1.85 (0.36) ***	−1.52 (0.27) ***	−1.50 (0.27) ***	−0.36 (0.13) **	−0.35 (0.13) **
(0 = non-single, 1 = single)						
Self-report	0.02 (0.02)		0.01 (0.02)		0.01 (0.01)	
psychopathy						
Factor 1—Egocentric	0.04 (0.04)		0.01 (0.03)		0.02 (0.02)	
Factor 2—Antisocial	0.17 (0.08) **		0.14 (0.06) *		−0.03 (0.03)	
Factor 3—Callous	0.22 (0.07) ***		0.19 (0.05) ***		0.04 (0.02)	
Psychosocial risk factors						
Impulsivity	0.02 (0.07)	−0.08 (0.08)	0.06 (0.05)	−0.03 (0.06)	0.04 (0.03)	0.05 (0.03) *
Sexual desire	0.11 (0.02) ***	0.10 (0.02) ***	0.08 (0.01) ***	0.07 (0.01) ***	0.03 (0.01) ***	0.03 (0.01) ***
Constant	3.68 (2.22) *	1.28 (2.13) *	3.36 (1.63) *	1.25 (1.56) *	0.32 (0.77) *	0.02 (0.75) *
Adjusted R^2^	0.17	0.19	0.18	0.20	0.10	0.10
*F*	21.77 ***	19.02 ***	23.36 ***	21.14 ***	11.99 ***	9.73 ***

Notes: Unstandardized beta (B) and standard error (SE). * *p* < 0.05, ** *p* < 0.01, *** *p* < 0.001.

## Data Availability

The data presented in this study are available on request from the corresponding author.
